# Identification and Characterization of a Bacteriocin from the Newly Isolated *Bacillus subtilis* HD15 with Inhibitory Effects against *Bacillus cereus*

**DOI:** 10.4014/jmb.2208.08006

**Published:** 2022-10-14

**Authors:** Sung Wook Hong, Jong-Hui Kim, Hyun A Cha, Kun Sub Chung, Hyo Ju Bae, Won Seo Park, Jun-Sang Ham, Beom-Young Park, Mi-Hwa Oh

**Affiliations:** 1 National Institute of Animal Science, Rural Development Administration, Wanju 55365, Republic of Korea; 2 Technology Innovation Research Division, World Institute of Kimchi, Gwangju 61755, Republic of Korea; 3 Division of Biological Science and Technology, Yonsei University, Wonju 26493, Republic of Korea

**Keywords:** Bacteriocin, *Bacillus subtilis*, antimicrobial activity, subtilosin

## Abstract

Natural antimicrobial substances are needed as alternatives to synthetic antimicrobials to protect against foodborne pathogens. In this study, a bacteriocin-producing bacterium, *Bacillus subtilis* HD15, was isolated from *doenjang*, a traditional Korean fermented soybean paste. We sequenced the complete genome of *B. subtilis* HD15. This genome size was 4,173,431 bp with a G + C content of of 43.58%, 4,305 genes, and 4,222 protein-coding genes with predicted functions, including a subtilosin A gene cluster. The bacteriocin was purified by ammonium sulfate precipitation, Diethylaminoethanol-Sepharose chromatography, and Sephacryl gel filtration, with 12.4-fold purification and 26.2% yield, respectively. The purified protein had a molecular weight of 3.6 kDa. The N-terminal amino acid sequence showed the highest similarity to *Bacillus subtilis* 168 subtilosin A (78%) but only 68% similarity to *B. tequilensis* subtilosin proteins, indicating that the antimicrobial substance isolated from *B. subtilis* HD15 is a novel bacteriocin related to subtilosin A. The purified protein from *B. subtilis* HD15 exhibited high antimicrobial activity against *Listeria monocytogenes* and *Bacillus cereus*. It showed stable activity in the range 0–70°C and pH 2–10 and was completely inhibited by protease, proteinase K, and pronase E treatment, suggesting that it is a proteinaceous substance. These findings support the potential industrial applications of the novel bacteriocin purified from *B. subtilis* HD15.

## Introduction

Foodborne pathogens are a major public health threat and an economic burden in the food industry and society in general [1]. *Bacillus cereus* and *Listeria monocytogenes* are ubiquitous microorganisms in the natural environment [2, 3] and are well-known foodborne pathogens that cause emesis and diarrhea [4]. *L. monocytogenes* causes listeriosis, a significant health hazard to newborns, pregnant women, and elderly individuals [5].

Preservatives, either synthetic or natural, are added to food to prevent spoilage and poisoning by foodborne spoilage and pathogenic bacteria. Recently, there has been a trend toward avoiding synthetic preservatives to address safety concerns [6] and to meet increasing consumer demand for natural preservatives, including organic acids, plant extracts, and antimicrobial substances produced by microorganisms, such as proteins and peptides [7]. These substances can be degraded by digestive enzymes, which are later absorbed by the body [8]. For example, the bacteriocin nisin produced by *Lactococcus lactis* was approved by the United States Food and Drug Administration in 1998 and has since been used as a preservative in processed cheese [9].

Antimicrobial substances produced by *Bacillus* species have more diverse characteristics and a broader range of activities than those of substances produced by lactic acid bacteria [10, 11]. However, the current classification system for antimicrobial peptides generated by ribosomal synthesis is based solely on bacteriocins produced by lactic acid bacteria [12]. These can be categorized into four classes. Class I comprises small peptides (<5 kDa) containing unusual amino acids (*e.g.*, lactionine and 3-methyllanthionine), including subtilin, sublancin 168, and subtilosin A, which are generated as polypeptides that undergo post-translational modifications [12-14]. Class II (non-lantibiotic) bacteriocins are relatively small (<10 kDa), heat-stable compounds that include pediocin-like peptides (class IIa), two-peptide complexes (class IIb), circular bacteriocins (class IIc), and non-pediocin-like bacteriocins (class IId), which are synthesized on ribosomes and do not undergo post-translational modifications. Examples of class II bacteriocins include coagulin, thurincin H, and lichenin [15, 16, 17]. Class III antimicrobials are bacteriocins that have a molecular weight greater than 30 kDa and are heat-sensitive, such as megacin A. Class IV bacteriocins are complex peptides containing essential lipid or carbohydrate moieties for their activity [12-14].

Many antimicrobial peptides have not been classified owing to a lack of DNA and protein sequence information; these are referred to as bacteriocin-like inhibitory substances [12]. Those produced by *B. subtilis* LFB112 disrupt both gram-positive and gram-negative bacteria, whereas substances produced by *B. subtilis* MJP1 have antimicrobial activity against gram-positive bacteria and fungi [18]. Given their broad range of activities, antimicrobial substances produced by *Bacillus* species have applications in many industries, in addition to the food industry [19].

Korean traditional fermented foods, including *doenjang*, *cheonggukjang*, gochujang, and soybean, are good resources for isolating beneficial microorganisms harboring antimicrobial properties to be used as starter cultures. This study screened four traditional fermented food products for the presence of bacteriocin-producing microorganisms capable of killing the bacteria *Bacillus cereus*. In addition, growth inhibition of *B. cereus* using HD15 present in the Korean fermented food, *doenjang*, was investigated. The antimicrobial substance produced by HD15 was purified and characterized to assess its value as a novel bio-preservative for foods.

## Materials and Methods

### Isolation and Culture of Microorganisms

Microorganisms were isolated from traditionally produced *doenjang* (a traditional Korean fermented soybean paste), *meju* (a brick of dried fermented soybeans), *ganjang* (a Korean soy sauce made from fermented soybeans), and *cheonggukjang* (a traditional Korean fermented soybean). Separated fractions of each sample were mixed with sterile 0.85% NaCl solution at a 1:9 ratio for 10 min with a homogenizer (Seward Laboratory Systems, USA) and diluted 10-fold in sterile 0.85% NaCl. A 100 μl volume of the suspension was smeared on a tryptic soy agar plate (TSA; Difco, USA) and incubated at 37°C for 14 h.

### Evaluation of Antimicrobial Effects

Antimicrobial activity against several gram-positive and gram-negative bacteria was assessed using the agar well diffusion method [20], with some modifications. Cultures were incubated in tryptic soy medium at 37°C for 60 h. Cell-free supernatants were prepared by centrifugation (3,800 ×*g*, 4°C, 30 min) and used as an antagonistic substance. Each pathogen at 7.0 log CFU/ml was spread on a TSA plate. An 8-mm-diameter well was created using a cork borer on the agar plate, and 50 μl of the cell-free supernatant was added to the wells. The plates were left undisturbed for a few hours to allow the supernatant to diffuse into the agar and then cultured at 37°C for 18 h. The diameter of the resultant growth inhibition zone was measured as a measure of antimicrobial activity.

### PCR Amplification and Sequencing of the 16S rRNA and rpoB Genes

The nearly full-length 16S rRNA from the selected genomic DNA was amplified by PCR with combinations of primers (338R, GCTGCCTCCCGTAGGAGT; 926F, AAACTCAAAGGAATTGACGG; 1088R, GCTCGTTGC GGGACTTAACC; and 1492R, GGATACCTTGTTACGACTT). To amplify the *rpoB* gene, the primers *rpoB*1206 (ATC GAA ACG CCT GAA GGT CCA AAC AT) and *rpoB*R3202 (ACA CCC TTG TTA CCG TGA CGA CC) were used. The two genes were amplified under the same PCR conditions with the following cycling program: 95°C for 5 min; 35 cycles of 94°C for 45 s, 52°C for 45 s, and 72°C for 1 min; and a final extension at 72°C for 5 min. After the PCR products were separated by 1% agarose gel electrophoresis, samples were purified using the Genomic DNA Clean & Concentrator-10 Kit (Zymo Research, USA), according to the manufacturer's instructions. The purified PCR product was sequenced on an ABI PRISM 3730XL Analyzer (Applied Biosystems, USA). All 16S rRNA and *rpoB* gene sequences were assembled to obtain the full-length sequences, which were searched against the NCBI GenBank (http://www.ncbi.nlm.nih.gov) using the Basic Local Alignment Search Tool (BLAST) (http://blast.ncbi.nlm.nih.gov/Blast.cgi) for taxonomic classification.

### Genome Sequencing and Annotation

Genomic DNA was extracted using a Wizard Genomic DNA Isolation Kit (Promega, USA). The genome of strain HD15 was subjected to de novo sequencing using the Pacific Biosciences (PacBio) RS II Single-molecule Real-time (SMRT) Cell Sequencing Technology (Macrogen, Korea). De novo assembly was performed using RS HGAP assembly version 3.0 [21]. The genome sequence was annotated using the RAST server and BlastKOALA (KEGG Orthology and Links Annotation). Gene prediction was carried out using Prodigal, and the predicted proteins were searched for similarity against the UniProt protein database using Blastp, followed by pathway identification using the KEGG server.

### Purification of Bacteriocins

The selected isolate was cultured in 500 ml of TSB at 1% (v/v) for 60 h at 37°C with shaking. The culture was centrifuged at 10,000 ×*g* for 30 min, and the supernatant was collected for the purification of the bacteriocin. Ammonium sulfate (Junsei Chemical Co., Japan) was added to the culture supernatant at 80% saturation, followed by incubation at 4°C for 12 h. After centrifugation, the precipitate was dissolved in 10 mM Tris-HCl and the solution was dialyzed (molecular weight cut-off: 6–8 kDa; Thermo Fisher Scientific, USA) at 4°C for 12 h using 10 mM Tris-HCl (pH 8). All purification steps were performed at 4°C to prevent protein denaturation.

A 2.5 cm × 40 cm anion-exchange Diethylaminoethyl-Sepharose Fast-Flow Column (Pharmacia Biotech, Sweden) was equilibrated with 10 mM Tris-HCl, and the ammonium sulfate-precipitated bacteriocin was injected into the column along with the buffer at a flow rate of 1 ml/min. A linear gradient of 0–1 M NaCl in buffer was used for elution, and 5 ml fractions were collected every minute. The protein content in each fraction was measured on a spectrometer at a wavelength of 280 nm, and fractions with antimicrobial activity were combined and lyophilized. Bacteriocin was fractionated by ion-exchange chromatography, and gel chromatography was performed using a 1.5 cm × 96 cm Sephacryl S-200HR Column (Pharmacia Biotech) equilibrated with 10 mM Tris-HCl buffer and eluted at a flow rate of 0.5 ml/min. The protein content in each 3 ml fraction was measured using a spectrometer at a wavelength of 280 nm, and fractions with antimicrobial activity were combined and lyophilized.

### Quantification of Protein Content in the Bacteriocin Solution

The protein content in the bacteriocin solution was measured using the modified Lowry method [22]. A 50 μl volume of bacteriocin was mixed with 550 μl of biuret reagent (0.75 mM cupric sulfate and 94 mM sodium hydroxide) and incubated for 10 min at 25°C. A 25 μl volume of Folin–Ciocalteu’s phenol reagent (Sigma-Aldrich, USA) was then added, followed by incubation for 30 min at 25°C. Absorbance was measured at 725 nm using a VersaMax ELISA Microplate Reader (Molecular Devices, USA), and a standard curve was constructed using bovine serum albumin (Sigma-Aldrich). Antimicrobial activity was measured as arbitrary units per milliliter of purified microbial culture using serial 2-fold dilutions of the antimicrobial substance. The reciprocal of the maximum dilution that resulted in a transparent zone was considered the activity in AU. AU/mL was calculated by multiplying AU by the dilution factor.

### Measurement of Molecular Weight of Bacteriocin

The molecular weight of bacteriocin was determined by tricine sodium dodecyl sulfate-polyacrylamide gel electrophoresis (SDS-PAGE; Bio-Rad, USA) at 100 V for 5 h on a 20% polyacrylamide gel with an ultra-low-range molecular weight marker (1,060–26,600 Da; Sigma-Aldrich), followed by silver staining (Amersham Biosciences, Sweden). Direct detection was then performed to determine whether the protein bands corresponded to bacteriocin [23].

### N-Terminal Amino Acid Sequencing

Purified bacteriocin was separated by tricine-SDS-PAGE and transferred at 17 V for 40 min to a polyvinylidene difluoride membrane (Bio-Rad) equilibrated in buffer composed of 100 ml of 10× transfer buffer (30.3 g of Tris, 144.2 g of glycine, and 1 L of distilled water, pH 8.3), 200 ml of methanol, and 700 ml of distilled water. The membrane was stained with Coomassie Brilliant Blue, destained with methanol, and then dried to confirm bacteriocin staining. The sequential identification of peptides using a protein/peptide sequencer (model 494; Applied Biosystems) was performed at the Korea Basic Science Institute in Korea, according to the method described by Edman and Begg [24].

### Bacteriocin Stability

To evaluate the pH stability of the antimicrobial substance, the buffers were prepared with 0.1 M glycine-HCl buffer (pH between 2 and 4), 0.1 M sodium acetate buffer (pH between 4 and 6), 0.1 M sodium phosphate buffer (pH between 6 and 8), and 0.1 M Tris-HCl buffer (pH between 8 and 10). The antimicrobial substance was mixed with buffer at a ratio of 1:15 and incubated at 37°C for 12 h, and relative antimicrobial activity was measured. To evaluate temperature stability, purified bacteriocin was incubated at 0°C, 20°C, 40°C, 60°C, 70°C, 80°C, or 90°C for 12 h, and at 100°C for 1 h. Relative antimicrobial activity was assessed using the agar well diffusion method, and the zone of inhibition was measured in millimeters. To assess the effect of various enzymes on antimicrobial activity, lysozyme (E.C. 3.2.1.17), α-amylase (E.C. 3.2.1.1), lipase (E.C. 3.1.1.3), protease (E.C. 3.4.24.31), and proteinase K (E.C. 3.4.21.64) (all from Sigma-Aldrich) and pronase E (E.C. 3.4.24.4; Merck Millipore, USA) were prepared in sodium phosphate buffer (pH 7.0) at a final concentration of 4 mg/ml. Purified bacteriocin was mixed with each enzyme at 2 mg/ml and incubated at 37°C for 30 min, and the relative antimicrobial activity was measured.

### Nucleotide Sequence Accession Numbers

The subtilosin gene cluster nucleotide sequence reported here has been deposited in the EMBL nucleotide sequence database under the accession number AJ430547. This whole-genome shotgun project of *B. subtilis* HD15 was deposited in DDBJ/EMBL/GenBank under accession no. CP080508.

## Results and Discussion

### Isolation and Identification of Isolates with Antimicrobial Activity

**Fig. 1:**
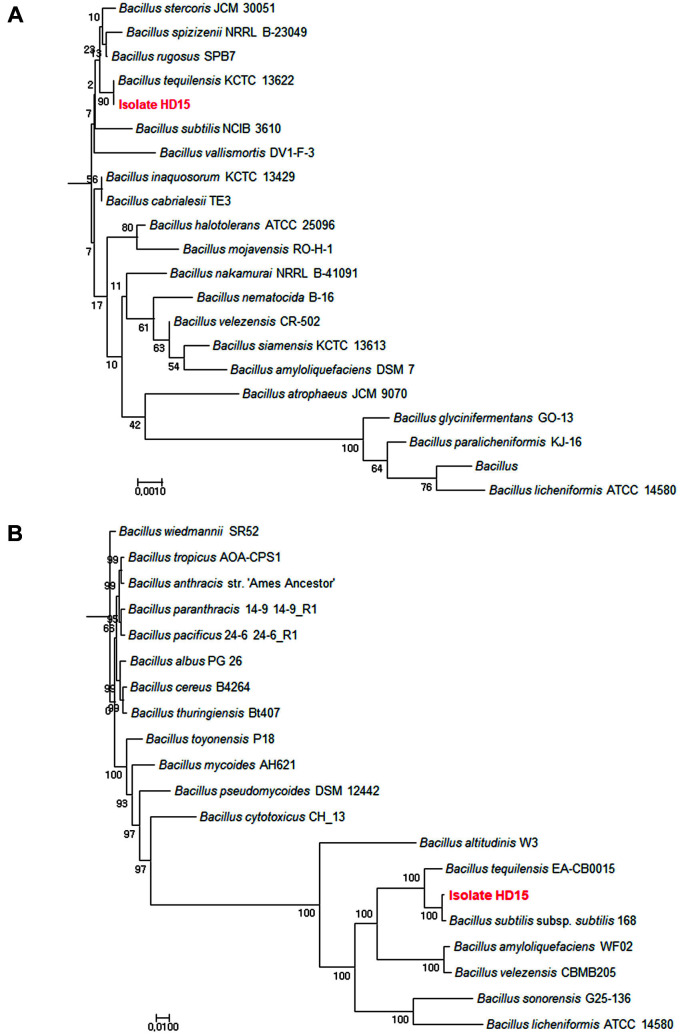
Phylogenetic analysis of isolate HD15 based on 16S rRNA (**A**) and *rpoB* (**B**) gene homology. Trees were constructed by the minimum evolution method using the MEGA 4 package. The number on each branch indicates the percentage of 1,000 replicates that includes the branch. Sequences determined in this study are shown in bold. Scale bar: 0.005 substitutions per site using the Jukes–Cantor model.

A total of 900 strains were isolated from various fermented soybean food products. Colonies that grew on TSA were tested for antimicrobial activity against *B. cereus*. The isolate exhibiting the highest antimicrobial activity (inhibitory zone, 17.02 ± 1.04 mm) was selected for further analyses (Table 1). We constructed two separate phylogenetic trees based on 16S rRNA and partial *rpoB* gene sequences. The isolate shared 100% identity with *Bacillus tequilensis* based on full-length 16S rDNA sequences (Fig. 1A). The *rpoB* gene showed a sequence similarity of 97% with the partial *rpoB* genes of *B. tequilensis* and *B. subtilis* subsp. subtilis. The constructed tree had high bootstrap values (Fig. 1B). Isolate HD15 and the position of these bacteria in the phylogenetic tree confirmed that the species are synonymous. The isolate was designated *B. subtilis* HD15 and was deposited in the Korea Culture Center of Microorganisms (KCCM) under the accession number KCCM 91944.

### General Genomic Features of *Bacillus subtilis* HD15

**Fig. 2:**
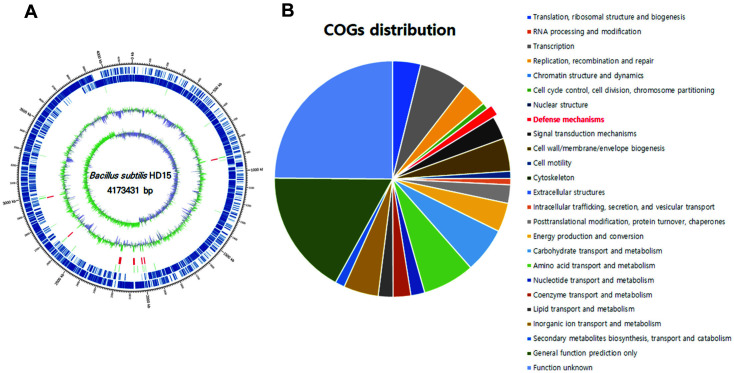
Genomic features of the chromosome of *B. subtilis* HD15. **A**, Circular genome maps of *B. subtilis* HD15 chromosome; **B**, Proportion of genes enriched in the Clusters of Orthologous Groups (COG) categories.

To investigate antibacterial factors, we conducted whole genome sequencing of *B. subtilis* HD15. Sequencing data were generated using the PacBio RS II SMRT cell sequencing technology. The general features of complete genome were 4,173,431 bp with a G + C content of 43.58%. The genome contained 4,305 CDSs, 86 tRNA genes, and 30 rRNA genes(data not shown). We also detected various genes related to antibacterial activity in the genome of *B. subtilis* HD15. We used a complete operon composed of eight genes to produce mature subtilosin A. It included *sboA*, which encodes the subtilosin prepeptide structural gene, and a seven-gene operon (albABCDEFG), which encodes the processing and immune genes for the antilisterial bacteriocin [25]. This leads to the complete expression of subtilosin. Furthermore, there was also an upstream gene that encodes a protein homologous to the bacteriocin UviB (uviB) (Fig. 2 and Table 2). The role of UviB, a second product of the uviAB operon, is currently undetermined. The UviB gene has been reported in the UV-inducible bacteriocin operon (uviA-uviB-bcn5) in the study of Rood and Cole [26]. However, comparing sequences with other proteins showed that UviB is similar to BhlA, a product of *Bacillus subtilis* phage SPβ, and the holin-protein [27].

### Purification of Bacteriocins

**Fig. 3:**
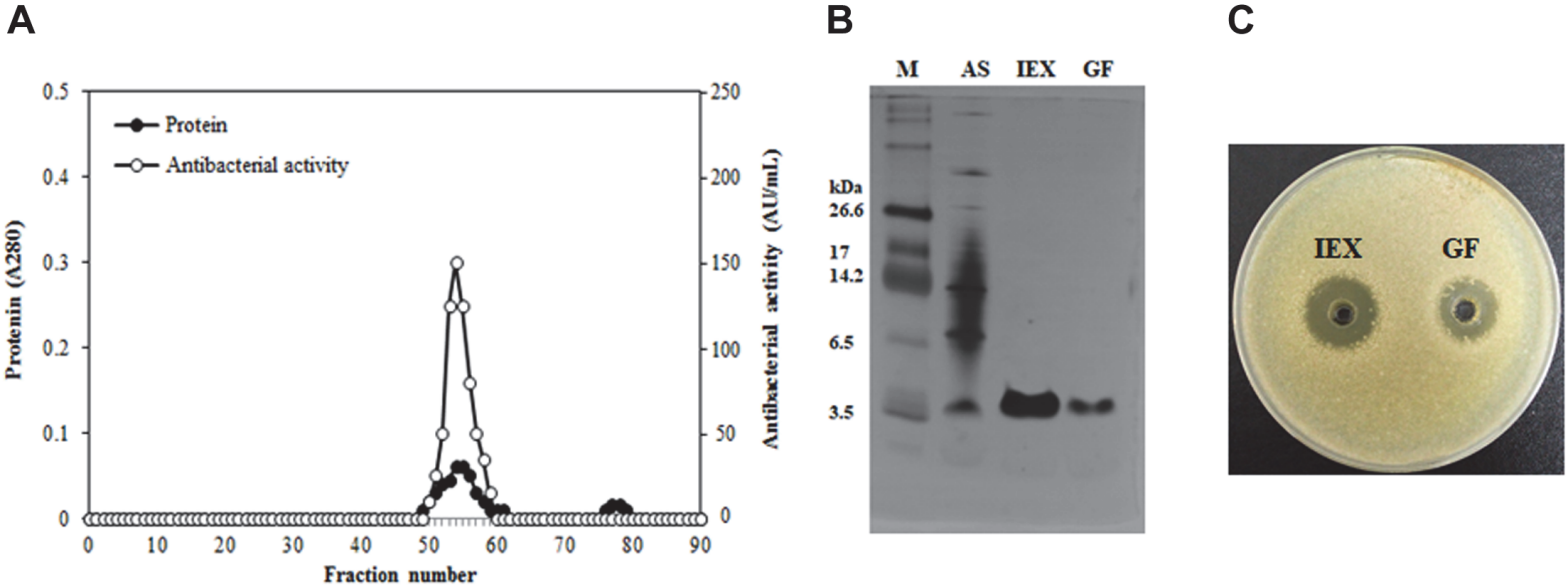
Analysis of antibacterial peptides from *B. subtilis* HD15. **A**, Chromatogram profile of gel filtration chromatography of bacteriocin, measured at 280 nm; **B**, Tricine SDS-PAGE analysis of purified bacteriocin; **C**, Antibacterial activity of purified bacteriocin as determined by the agar well diffusion test against *Bacillus cereus* KCCM 12667. Lane M, ultralow range molecular weight marker; lane AS, antimicrobial substance precipitated by 20–60% ammonium sulfate; lane IEX, antimicrobial substance eluted by Diethylaminoethyl-Sepharose FF ion exchange chromatography; lane GF, purified antimicrobial substance eluted by Sephacryl S-200HR.

Fractions 34–55 obtained by ion exchange chromatography showing antimicrobial activity were pooled and subjected to gel chromatography using Sephacryl. Fractions 49–61 showing antimicrobial activity were pooled and used as purified bacteriocin (Fig. 3A). Bacteriocin purification results are summarized in Table 3, showing 12.4-fold purification and a 26.2% yield. The molecular weight of the purified bacteriocin, determined by tricine SDS-PAGE, was 3.6 kDa. A single band corresponding to the purified bacteriocin was detected (Fig. 3B). Additionally, a clear zone surrounding the purified bacteriocin (Fig. 3C) was observed against *B. cereus* KCCM 12667.

Antimicrobial substances produced by *B. subtilis* with molecular weights of approximately 3.5 kDa include subtilin, subtilosin A, sublancin 168, and ericin S, which are class I bacteriocins. Subtilin is a pentacyclic peptide with a molecular weight of 3.3 kDa, and ericin S with a molecular weight of 3.4 kDa is highly similar to subtilin, differing by only four amino acid residues [28, 29]. Subtilosin has a molecular weight of 3.4 kDa and assumes a macrocyclic form following posttranslational modification [30]. Sublancin 168 is a 3.9-kDa glycopeptide that contains a lanthionine linked by two disulfide bonds [31]. Based on peptide size, the enzyme purified from *B. subtilis* HD15 was presumed to be a class I bacteriocin.

### Genetic Organization and Amino Acid Sequence Analysis of Purified Bacteriocins

**Fig. 4:**
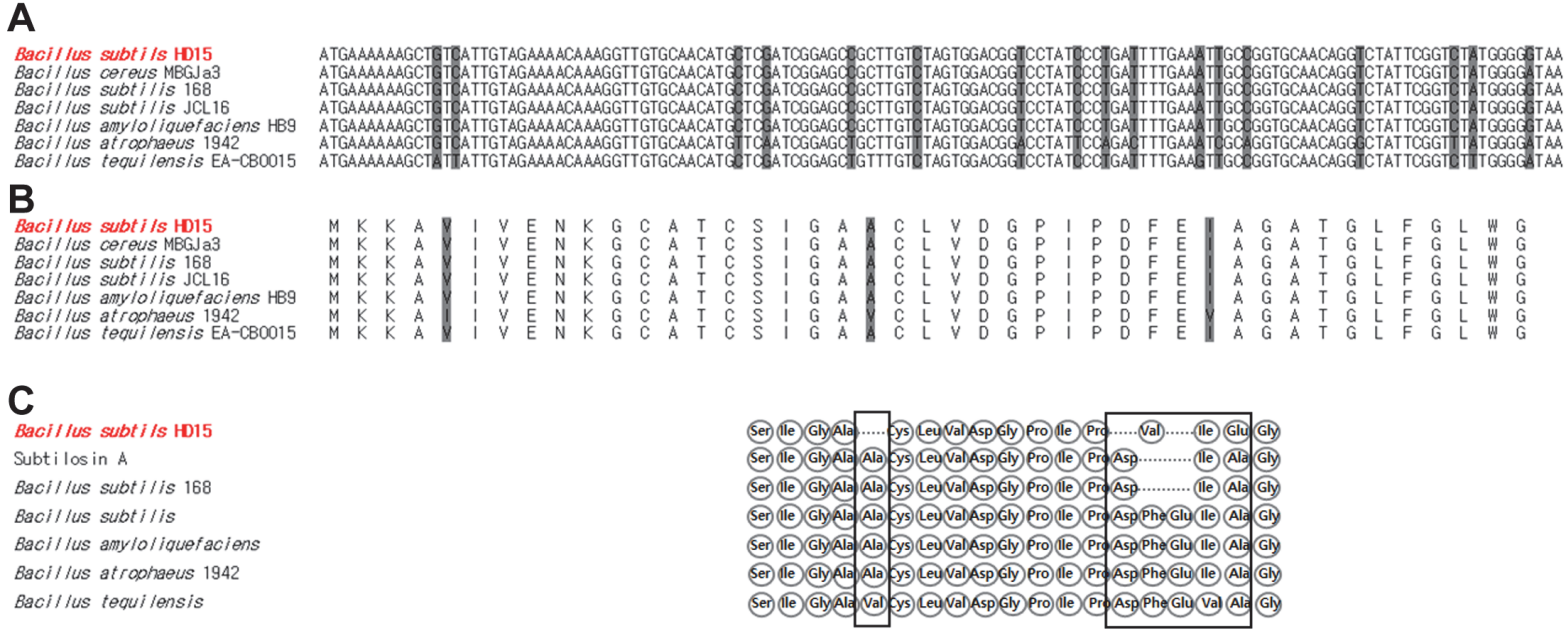
Comparison of Sbo alleles of seven *Bacillus* species. **A**, Sequence of the subtilosin A-encoding gene Sbo; **B**, Alignment of the derived amino acid sequences of the putative Sbo. Differences between the seven alleles are indicated by shading; **C**. Multiple sequence alignment of the N-terminal amino acid sequence of *B. subtilis* HD15.

The 43 amino acid sequence predicted from *sboA* of the bacteriocin from *B. subtilis* HD15 was Met-Lys-Lys-Ala-Val-Ile-Val-Glu-Asn-Lys-Gly-Cys-Ala-Thr-Cys-Ser-Ile-Gly-Ala-Ala-Cys-Leu-Val-Asp-Gly-Pro-Ile-Pro-Asp-Phe-Glu-Ile-Ala-Gly-Ala-Thr-Gly-Leu-Phe-Gly-Leu-Trp-Gly (Fig. 4A and Fig. 4B). A search of bacteriocin peptide databases revealed similarity with previously identified subtilosin A. The bacteriocin produced by *B. subtilis* HD15 showed 100% similarity to subtilosin A produced by five *Bacillus* species, including *B. cereus* MBGJa3 (GenBank Accession No. CP026523), *B. subtilis* 168 (GenBank Accession No. AL009126), *B. subtilis* JCL16 (GenBank Accession No. NZ_CP054177), *B. amyloliquefaciens* HB9 (GenBank Accession No. MT490213), *B. tequilensis* EA-CB0015 (GenBank Accession No. NZ_CP048852), and *B. atrophaeus* 1942 (GenBank Accession No. NC_014639, 93% similarity to subtilosin A).

The N-terminal amino acid sequence of the purified bacteriocin from *B. subtilis* HD15 was Ser-Ile-Gly-Ala-Cys-Leu-Val-Asp-Gly-Pro-Ile-Pro-Val-Ile-Glu-Gly, and the amino acid residues were experimentally confirmed using a protein/peptide sequencer. The N-terminus of the purified bacteriocin was used to generate a multiple sequence alignment with subtilosin sequences from the genus *Bacillus* in the *National Center* for Biotechnology Information database (Fig. 4C). Our purified bacteriocin sequence showed 78% and 68% similarity to those of subtilosin A (E.C. 3.4.21.62) from *B. subtilis* 168 (GenBank Accession No. 1109186A) and *B. tequilensis* (GenBank Accession No. WP_024713849.1). It was also 72.2% identical to subtilosin A from *B. subtilis* (GenBank Accession No. 1PXQ_A). Subtilosin production has been documented in several *B. subtilis* subspecies as well as in the closely related species *B. atrophaeus* [32] and *B. amyloliquefaciens* [33]. In addition, subtilosin A from *B. subtilis* was similar to the N-terminal amino acid sequence of subtilosin A produced by *Bacillus* species [34].

### Antimicrobial Activity Spectrum

The antimicrobial activities of *B. subtilis* HD15 bacteriocin against pathogenic bacteria are summarized in Table 4. Measurement of antimicrobial activity by the well diffusion method showed strong inhibition against gram-positive bacteria, such as *B. cereus* and *L. monocytogenes*, and no activity against gram-negative bacteria or *Staphylococcus aureus*, consistent with previous results for a bacteriocin from *B. subtilis* 168 [35].

Antimicrobial substances produced by gram-positive bacteria generally exhibit bacteriostatic activity. An antimicrobial substance (1,600 AU/ml) produced by *B. subtilis* H27 isolated from fermented soybean paste was toxic to *L. monocytogenes* after 12 h of treatment [36]. *B. subtilis* W42 isolated from *cheonggukjang* showed strong antimicrobial activity against *B. cereus* and *L. monocytogenes*; however, it showed no toxicity towards gram-negative bacteria [37]. Bacteriocins produced by most *Bacillus* species have no effect on gram-negative bacteria and inhibit only gram-positive species [38].

### Bacteriocin Stability

The bacteriocin in this study maintained 100% of its antimicrobial activity at pH 5–7; however, the activity decreased to 50% at pH 2, 80% at pH 3 to 9, and 30% at pH 10 (Table 5). It was previously reported that the activity of the antimicrobial substance produced by *B. subtilis* SC-8 was lower at pH 3 than at pH 4–10 [38]. Bacthuricin F4 produced by *Bacillus thuringiensis* showed 40% residual antimicrobial activity at pH 8, 10% at pH 9, and approximately 80% at pH 3 [38]. The buffer itself did not inhibit *B. cereus* growth (data not shown).

To measure temperature stability, purified bacteriocin was incubated at temperatures ranging from 0°C to 80°C for 12 h or at 100°C for 1 h before measuring antimicrobial activity. The activity was 100% after incubation at 0–50°C for 12 h but decreased to 70% after incubation at 70°C for 12 h. These results demonstrate that *B. subtilis* HD15 bacteriocin is stable over a range of temperatures. In contrast, the antimicrobial activity of a substance produced by *B. subtilis* SC-8 against *B. cereus* was lost after incubation at 80°C or 100°C for 1 h, and the activity against *L. monocytogenes* decreased by 50% after incubation at 60–80°C for 15 min and was reduced to 20% after incubation at 100°C for 10 min [36, 39]. The bacteriocin produced by *B. subtilis* HD15 was stable at extreme temperatures and pH, with more than 70% activity remaining after 12 h at 70°C and over a pH range between 3 and 9. This suggests that it can be adapted to a variety of applications, including but not limited to food preservation.

Since treatment with amylase and lipase had no effect on antibacterial activity (Table 5), we presumed that bacteriocin does not possess carbohydrate or lipid moieties or they are not essential for enzymatic activity. However, the antimicrobial activity of bacteriocin was lost upon exposure to proteolytic enzymes, such as protease, proteinase K, and pronase E (Table 5), confirming that the purified substance was proteinaceous [40]. The protein and peptide components of antibacterial bacteriocins produced by microorganisms is can be degraded by proteolytic enzymes in the digestive system. Based on these characteristics, we propose that bacteriocin purified from *B. subtilis* HD15 can be used as a natural food or feed preservative.

We obtained an isolate with high antimicrobial activity against *B. cereus* from *doenjang*. Molecular analysis revealed that the isolate was *B. subtilis* HD15. Purified bacteriocin exhibited excellent antimicrobial activity against both *L. monocytogenes* and *B. cereus*. The bacteriocin was stable up to 70°C and in the pH range of 2–10. The antimicrobial activity of bacteriocin was lost upon exposure to proteolytic enzymes, confirming its proteinaceous nature. Based on these characteristics, we propose that bacteriocin purified from *B. subtilis* HD15 is a promising bio-preservative and natural alternative to chemical preservatives in the food industry.

## Figures and Tables

**Table 1:** Antibacterial activity of bacterial isolates from various types of fermented soybean foods using agar plate diffusion experiment.

Isolates	Inhibiton zone (mm)	

	Diameter averaged value	Standard deviation
HC31	10.31	0.21
HD10	14.16	0.58
HD15	17.02	1.04
KC12	11.97	0.61
KR14	13.42	0.24

Isolates were cultured in TSB at 37°C for 24 h, and culture supernatants were tested for antimicrobial activity against *B. cereus* by the well diffusion method. HC and KC, HD, and KR were isolated from traditionally produced *cheonggukjang*, *doenjang*, and *meju*, respectively.

**Table 2:** Bacteriocin related genes present in *Bacillus subtilis* HD15.

Gene name	Gene locus number	Description
*sboA*	QYM62143	Subtilosin A
*albA*	QYM62145	Antilisterial bacteriocin subtilosin biosynthesis protein AlbA
*albB*	QYM62146	Antilisterial bacteriocin subtilosin biosynthesis protein AlbB
*albC*		Putative ABC transporter ATP-binding protein AlbC
*albD*	QYM62148	Antilisterial bacteriocin subtilosin biosynthesis protein AlbD
*albE*	QYM62657	Antilisterial bacteriocin subtilosin biosynthesis protein AlbE
*albF*		Putative zinc protease AlbF
*albG*	QYM62150	Antilisterial bacteriocin subtilosin biosynthesis protein AlbG
*uviB*		Bacteriocin UviB

**Table 3:** Summary of purification of bacteriocin from *Bacillus subtilis* HD15.

Steps	Total activity (AU)	Total protein (mg)	Specific activity (AU/mg)	Purification (fold)	Yield (%)
Culture supernatant	40,000	1,200	33.3	1	100
Ammonium sulfate precipitation	20,600	110.5	186.4	5.6	51.5
Diethylaminoethyl-sepharose FF column chromatography	15,200	51.2	296.9	8.9	38.0
Sephacryl S-200HR column chromatography	10,500	25.4	413.4	12.4	26.2

**Table 4:** Inhibitory spectrum of bacteriocin from *Bacillus subtilis* HD15.

Microorganism	Indicator species	Antibacterial activity
Gram-positive bacteria	*Bacillus cereus* KCCM 40152	+++
	*Listeria monocytogenes* ATCC 15313	+++
	*Staphylococcus aureus* ATCC 25923	−
Gram-negative bacteria	*Cronobacter sakazakii* KCTC2949	−
	*Escherichia coli* O157:H7 ATCC 43894	−
	*Pseudomonas aeruginosa* KCCM 12539	−
	*Salmonella choleraesuis* KCCM 40736	−
	*Salmonella enteritidis* CCARM 8206	−
	*Shigella sonnei* KCCM 41282	−
	*Shigella flexneri* KCCM 11937	−
	*Vibrio parahaemolyticus* KCCM 11965	−
	*Vibrio vulnificus* ATCC 29306	−

+++, Greater than 15 mm; −, no inhibition zone.

**Table 5:** Effect of pH, heat, and enzyme treatment on the antibacterial activity of *Bacillus subtilis* HD15.

Treatment	Relative activity (%)
pH	
2	50
3	80
4	95
5	100
6	100
7	100
8	95
9	80
10	30
Heat (temperature, °C)	
50	100
60	95
70	70
80	20
90	0
Enzymes	
α-Amylase	100
Lipase	100
Protease	0
Proteinase K	0
Pronase E	0
